# SPHK Inhibitors and Zoledronic Acid Suppress Osteoclastogenesis and Wear Particle-Induced Osteolysis

**DOI:** 10.3389/fphar.2021.794429

**Published:** 2022-02-14

**Authors:** Minghui Gu, Baiqi Pan, Weishen Chen, Hai Xu, Xiaoyu Wu, Xuantao Hu, Linli Zheng, Yongyu Ye, Qing Meng, Guoyan Xian, Ziji Zhang, Puyi Sheng

**Affiliations:** ^1^ Department of Joint Surgery, The First Affiliated Hospital of Sun Yat-sen University, Guangzhou, China; ^2^ Guangdong Provincial Key Laboratory of Orthopaedics and Traumatology, The First Affiliated Hospital of Sun Yat-sen University, Guangzhou, China; ^3^ Department of Radiology, The First Affiliated Hospital of Sun Yat-sen University, Guangzhou, China; ^4^ Department of Orthopaedics, Guangdong Provincial People’s Hospital, Guangdong Academy of Medical Sciences, Guangzhou, China; ^5^ Department of Orthopedics, Guizhou Orthopedics Hospital, Guiyang, China; ^6^ Université de Paris, CNRS, INSERM, B3OA, Paris, France

**Keywords:** SPHKs, osteoclast, aseptic prosthetic loosening, periprosthetic osteolysis, inflammation

## Abstract

**Background:** Inflammatory osteolysis induced by wear particles is the major cause of prosthetic loosening after artificial joint replacement, and its prevention and treatment are difficult worldwide. Our previous study confirmed that sphingosine kinases (SPHKs) are important mediators regulating the wear particle-induced macrophage inflammatory response. However, it is unclear whether SPHKs can modulate chronic inflammation and alleviate osteolysis. Zoledronic acid (ZA), an imidazole-containing bisphosphonate, directly affects osteoclasts and prevents bone mineral-related diseases. However, the effects of SPHK inhibitors and ZA used to treat periprosthetic osteolysis are unknown.

**Methods:** We applied tartrate-resistant acid phosphatase (TRAP) staining to evaluate bone destruction in the interface membranes of patients with aseptic loosening and a control group. A murine calvarial osteolysis model was used to examine the preventative effect of SPHK inhibitors and ZA on osteolysis. Micro-CT scanning, immunohistochemistry (IHC), and histomorphometric analysis were conducted to determine the variations in inflammatory osteolysis. The effects of different drug concentrations on cell viability were evaluated using the Cell Counting Kit-8 (CCK-8) assay. Real-time quantitative polymerase chain reaction (RT-qPCR) analysis was performed to confirm the reduced expression of osteoclast-specific genes after drug and titanium treatment. The osteoclast formation and functions of the drugs were analyzed using TRAP staining *in vivo* and *in vitro*. The effect of SPHKs/S1P-TRAF2-BECN1 signaling pathways was verified via RT-qPCR and tissue IHC.

**Results:** In this study, we found that SPHK inhibitors (ABC294640 and FTY720) combined with ZA decreased the degree of inflammatory osteolysis *in vivo*. However, ABC294640 and ZA suppressed osteoclast differentiation and osteoclast-specific genes *in vitro*. SPHKs regulate the inflammatory osteolysis induced by wear particles by increasing the expression of SPHKs/S1P-TRAF2-BECN1.

**Conclusion:** Our study revealed that wear particles could induce inflammatory osteolysis by upregulating SPHKs/S1P-TRAF2-BECN1 and SPHK inhibitors/ZA inhibit osteoclastogenesis in vitro and prevent inflammatory osteolysis in vivo, suggesting that SPHK inhibitors and ZA can be a new perspective and scientific basis for the prevention and treatment of prosthesis loosening.

## Introduction

Aseptic prosthesis loosening (APL) is one of the most common complications after artificial joint replacement and is the most critical cause of prosthesis (Learmonth et al., 2007). The incidence rate is approximately 10% in 10 years post operation (Gallo et al., 2014; Goodman and Gallo, 2019). However, the pathological mechanism and specific treatment of APL remain unclear, and most APL patients need revision surgery or multiple operations. Therefore, it is of clinical significance and educational value to investigate the internal mechanism of the occurrence and development of aseptic loosening of joint prostheses and explore the corresponding molecular targets, potential new drugs, and treatment strategies.

Studies have confirmed that inflammatory osteolysis caused by the activation of macrophages by the prosthesis wear particles in the body is the major reason for APL (Goodman et al., 2014; Pajarinen et al., 2014). Wear particles, primarily titanium (Ti) particles, stimulate macrophages and other cells to release cytokines, chemokines, and other pro-inflammatory substances (tumor necrosis factor-alpha [TNF-α] and interleukin-6 [IL-6]), which allow chronic inflammation to further induce osteoclast (OC) resorption and inhibit bone formation (Mbalaviele et al., 2017). These processes trigger periprosthetic osteolysis. During the development of inflammatory osteolysis, the homeostasis between OCs and osteoblasts is disrupted, and the normal structure of the bone tissue around the prosthesis cannot be maintained, leading to the sinking and displacement of the prosthesis, thereby causing APL (Purdue et al., 2007; Mbalaviele et al., 2017; Gao et al., 2018).

Sphingosine kinases (SPHKs) are conservative lipid kinases, and the SPHK signaling pathway is closely related to cell survival and plays an important role in the regulation of cell inflammation, proliferation, apoptosis, migration, and tumor metabolism (Edwards et al., 2006). SPHKs are divided into two subtypes, SPHK-1 and SPHK-2, that have different tissue distributions, subcellular localizations, and biological activities. Our previous studies have found that SPHKs are important mediators that regulate the inflammatory response of macrophages induced by wear particles. The upregulation of SPHKs enables macrophages to synthesize and continuously release inflammatory factors, causing local persistent inflammation and immune function damage, eventually leading to loosening of the prosthesis (Yang et al., 2018). SPHK2 generates sphingosine-1-phosphate (S1P) as an isoenzyme (Hait et al., 2009). Furthermore, recent studies have revealed that SPHK1 and S1P play important roles in OC genesis regulation (Ishii et al., 2009; Chung et al., 2014), providing a basis for exploring the therapeutic potential of SPHKs and S1P for inflammatory disease-associated bone destruction.

Studies have shown that SPHKs/S1P and TNF receptor-associated factor 2 (TRAF2) further dissociate the Beclin-1 (BECN1) complex by activating mitogen-activated protein kinases (MAPKs), thereby regulating the flux and level of autophagy (Schenten et al., 2011; Ruspi et al., 2014; Liu et al., 2017). The autophagy process regulates the survival and mineralization of osteoblasts (OBs) and the bone resorption function of OCs (Wang et al., 2015; Wang et al., 2017; Camuzard et al., 2019). Our previous study revealed that autophagy regulation by macrophages is closely related to the progress of inflammatory osteolysis in APL (Xian et al., 2020; Chen et al., 2021). The present study suggests that BECN1 contributes to receptor activator of nuclear factor-kappa B ligand (RANKL)-induced OC differentiation as a molecular link that mediates mitochondrial reactive oxygen species (mROS) and nuclear factor of activated T-cell cytoplasmic 1 (NFATc-1) expression. Thus, BECN1 plays a role in OC genesis (Chung et al., 2014). Previous studies have shown that SPHK inhibitors (FTY720, ABC294640, and PF-543) can regulate S1P by inhibiting SPHK-1/2 and maybe potential therapeutic drugs for autoimmune diseases (Pyne et al., 2017). Zoledronic acid (ZA) is an imidazole-containing bisphosphonate with a direct suppressive effect on OCs and the prevention of bone mineral-related diseases. However, the effects of SPHK inhibitors and ZA used as a treatment for preventing periprosthetic osteolysis are still unclear.

However, the related effects and mechanisms of SPHKs have not been investigated in prosthesis loosening and periprosthetic osteolysis mediated by wear particles. Based on our study, SPHKs play a role in the Ti particle-induced production of pro-inflammatory cytokines (IL-6 and TNF-α) in macrophages, thereby prolonging chronic inflammation and reinforcing the autoimmune reactions in the microenvironment surrounding the implant. This breaks the balance between bone formation or resorption, triggering APL. In this study, we show that wear particles upregulated the expression of SPHKs through the SPHKs/S1P-TRAF2-BECN1 signaling pathway, thereby contributing to inflammatory osteolysis. ZA and SPHK inhibitors (FTY720/ABC294640) reduce inflammatory osteolysis induced by wear particles.

The findings demonstrate the function and role of SPHKs in wear particle-induced inflammation and OC genesis. Thus, enhancing the knowledge about the effect of SPHKs in inflammatory osteolysis could be a key to the development of therapeutic interventions for aseptic implant loosening.

## Materials and Methods

### Patients and Specimens

The specimens were attained from patients admitted to the Joint Surgery Department of the First Affiliated Hospital of Sun Yat-Sen University from February 2009 to May 2021. All experiments were performed in accordance with the Guidelines of the Declaration of Helsinki, and experiments were approved by the Ethics Committee of the First Affiliated Hospital of Sun Yat-Sen University (ethical approval number: [2021]676). We got all specimens after the consent of the participants. In the APL group, the patients were confirmed to be diagnosed with aseptic loosening of their prosthesis, and the specimens of every patient who underwent revision were tested pathologically or subjected to bacterial fungus culture to exclude them from the periprosthesis infection of the joint (PJI). Periprosthetic interface membranes of patients (three males and two females) who were diagnosed with aseptic loosening during revision of total hip arthroplasty (THA) were collected. Except for clinical and imaging evidence, cultures for bacteria and fungi were negative in aseptic loosening. The synovial tissue was collected from patients with primary THA due to osteoarthritis or amputation with normal joint as the control group. Cases with either immune system or connective tissue disease were excluded. There were no significant differences in sex and age between the two groups. The chi-square test or Fisher test was used to analyze the difference on gender between the two groups. The difference was regarded as statistically significant when the *p*-value of continuity correction was <0.05. The specimens were immediately fixed in 4% paraformaldehyde for 24 h, dehydrated with ethanol, and embedded in paraffin. The sections were then stained and photographed under a light microscope.

### Preparation of Drug and Ti Particle

The Ti particles used in this study were purchased from Alfa Aesar (catalogue no. 00681, Ward Hill, MA, United States). The average diameter of the Ti particles is 3.2 ± 2.7 μm. The Ti particle storage solution is washed with 70% ethanol and autoclaved to remove endotoxins, according to the previously published methods (Yang et al., 2018; Xian et al., 2020; Chen et al., 2021). After sterility is ensured, the Limulus assistant kit (Xiamen Houshiji, Fujian, China) is used to determine the endotoxin, and endotoxins <0.01 Eu can be used for experiments *in vivo* and *in vitro*. PBS was removed by centrifugation (10,000 g/min) and was placed in an oven to generate a dry powder of Ti particles. The storage temperature was 5°C. SPHK inhibitors (ABC294640 and FTY720) and ZA were purchased from Selleck Chemicals (MA, United States). The concentrations of ABC294640 (20 μM) and ZA (1 μM) in this study have been confirmed in the Cell Counting Kit-8 (CCK-8) test to ensure that it will not significantly affect the survival of RAW 264.7 cells.

### Animals Experiments

All animal procedures were performed in accordance with the Guidelines for Care and Use of Laboratory Animals of Sun Yat-Sen University, and experiments were approved by the Institutional Animal Care and Use Committee of Sun Yat-Sen University. Thirty-six 7-week-old C57BL/6 male mice with an average weight of 21 ± 4 g were purchased from Jicui Company, Jiangsu. Then the calvarial model of wear particle-induced osteolysis was established according to the literature (Shao et al., 2015). The mice were randomly divided into seven groups (number of sample: *n* = 6 per group) as follows: Sham, Ti particles (Ti), Ti particles with ABC294640 (ABC294640 + Ti), Ti particles with FTY720 (FTY720 + Ti), Ti particles with ZA (ZA + Ti), Ti particles with ABC294640 and ZA (ABC294640 + ZA + Ti), and Ti particles with FTY720 and ZA (FTY720 + ZA + Ti). Before the surgery, 10% (w/v) sodium pentobarbital at a dose of 70 μl/20 g was utilized to inject mice intraperitoneally. After the mice were fully anesthetized, a sagittal incision about 1 cm long was made through the midpoint of the outer ear, and the periosteum was separated from the skull. The sham operation group has no other treatment. In addition, 20 mg of dry Ti powder was placed under the periosteum at the seam of the exposed calvaria. The skin is sutured intermittently to prevent the Ti particles from overflowing. Mice in the Ti particles with ABC294640/FTY720/ZA (ABC294640 + Ti, FTY720 + Ti, and ZA + Ti) group and Ti particles with ABC294640/FTY720 and ZA (ABC294640 + ZA + Ti and FTY720 + ZA + Ti) group were injected intraperitoneally with ABC294640 (25 mg/kg/day)/FTY720 (10 mg/kg/day)/ZA (0.15 mg/kg/day), respectively, for 2 weeks. The dosage of the SPHK inhibitors (ABC294640, FTY720, and ZA) for intraperitoneal injection refers to published literatures (Croucher et al., 2003; French et al., 2010; Hu et al., 2011). On day 14, the mice were euthanized, and the calvarial samples were fixed in 4% paraformaldehyde for micro-CT scanning and immunohistochemistry (IHC).

### Micro-CT Scanning

In order to eliminate metal artifacts, the wear particles were removed from the fixed calvarial samples. An Inveon micro-CT scanner (Siemens Inveon™, Munich, Germany) was utilized to scan calvarial samples in moderate resolution. The scanning protocol was set at an isometric resolution of 33 μm, with X-ray energy settings of 80 kV and 500 μA. For three-dimensional (3D) image reconstruction with Inveon Research Workplace 3.0 software, the same region of interest (ROI) around the midline suture was selected for further quantitative analysis. The bone volume-to-tissue volume ratio (BV/TV) was measured using the MicroView software (GE Medical Systems, Waukesha, WI, United States), and the number of pores of each sample was measured using Image-Pro Plus 6.0 software.

### Immunohistochemistry Analysis

After the micro-CT analysis was completed, decalcification was carried out in 10% ethylenediaminetetraacetic acid solution at 4°C for 4 days, followed by paraffin embedding. Histological sections (5 mm thick) were cut and prepared in the coronal plane for tartrate-resistant acid phosphatase (TRAP) staining, hematoxylin and eosin (H&E) staining, and detection of TNF-α (GB11188, Servicebio, China) and IL-6 (GB11117, Servicebio, China). Detection of SPHKs, BECN1, and TRAF2 was performed on the specimens. The number of TRAP-positive multinucleated OCs was assessed in each calvaria and specimens. Stained sections were observed and photographed under an optical microscope (Leica Aperio CS2, Germany). The Image-Pro Plus 6.0 software was used to analyze the ROI of each section.

### Cell Culture and OC Differentiation

Dulbecco’s modified Eagle medium containing 10% FBS was used to culture RAW264.7 mouse macrophages (American Type Culture Collection, Rockville, MD, United States). RAW264.7 cells were fully scraped off from the bottom of the culture bottle, centrifuged at 1,500 *g* for 5 min, and then resuspended in α-MEM containing 10% FBS. The cells were seeded into a 12-well plate at a density of 2 × 10^4^ cells per milliliter and incubated at 37°C with 5% CO_2_. According to a previous article (Chen et al., 2021), the induction medium (30 ng/ml RANKL, α-MEM) was changed once a day after the cells adhered for 24 h. OCs in the fusion phase began to appear on the third to fourth days. On the fifth day, OCs were identified by TRAP staining, and cells with more than three nuclei were defined as TRAP-positive cells.

### TRAP Staining

After the generation of OCs as mentioned above, cells were washed with PBS and fixed with 4% paraformaldehyde. To determine the TRAP + OCs, TRAP + cells were analyzed using the TRAP staining kit (catalogue no. 387-A, Sigma-Aldrich, CA, United States), following the manufacturer’s instructions. Cells for TRAP staining containing red granular material and with more than three nuclei were defined as TRAP-positive cells. The cells were examined under a microscope (Leica DM4000 B) and counted.

### Real-Time Quantitative Polymerase Chain Reaction

Total RNA was extracted using TRIzol universal reagent (Tian Gen, Beijing, China) and reverse-transcribed into cDNA using the PrimeScript RT Master Mix Kit (Takara, RR036A, Japan). Real-time quantitative PCR (RT-qPCR) was performed in the CFX96 system (Bio-Rad, CA, United States) using the TB Green Premix Ex Taq II Kit (Takara, RR820A, Japan). All mouse primer sequences are listed in Table 1. Relative expression levels of genes were calculated using the 2^−ΔΔCt^ method.

**TABLE 1:** Murine primer sequence set for qPCR.

**Gene**	**Forward primer**	**Reverse primer**
Becn1	5′-CCG​GGA​AGT​AGC​TGA​AGA-3′	5′-CTC​GTG​TCC​AGT​TTC​AGA-3′
NFatc-1	5′-GAC​CCG​GAG​TTC​GAC​TTC​G-3′	5′-TGA​CAC​TAG​GGG​ACA​CAT​AAC​TG-3′
Traf2	5′-CGG​AGT​GTC​CTG​CAT​GTA​AA -3′	5′-TGC​ATG​CTC​TAA​CAT​GGT​CC-3′
Gapdh	5′-AGG​TCG​GTG​TGA​ACG​GAT​TTG-3′	5′-TGT​AGA​CCA​TGT​AGT​TGA​GGT​CA-3′
S1p	5′-TCC​TCT​ACT​GCA​GGA​TCT​AC-3′	5′-ACT​CTG​CTT​TGT​ACA​GGA​TG-3′

### Overexpression Plasmid Cell Transfection

The day before transfection, the cell line was passaged until it reached 70%–80% confluence. Lipofectamine 2000 (Invitrogen, cat. no. 11668019) transfection reagent was used for transfection, and Opti-MEM (Invitrogen, cat. no. 31985070) was used as culture medium. With a six-well plate, 3 μg plasmid (SPHK-overexpressing pcDNATM3.1 vectors) was added to each well and then diluted with 100 μl Opti-MEM medium as solution A; 9 μl Lipofectamine 3000 was taken and dissolved in Opti-MEM medium as solution B, which was mixed for 5 min; solutions A and B were mixed and added to the cell culture plate after letting the mixture stand for 20 min. The above operation is the dosage per hole. After incubation for 4–6 h, the medium was changed to cell growth medium.

### Statistical Analysis

Data of quantitative experiments were expressed as the mean ± standard error of the mean (SEM). A comparison of paired groups was performed using Student’s *t*-test or Kruskal–Wallis test. Continuous variables of normal distribution were analyzed by one-way analysis of variance (ANOVA). Statistical analysis was performed using SPSS 19.0 software (SPSS, Chicago, United States) and GraphPad Prism version 8.0 (GraphPad software, San Diego, CA). The difference was regarded as statistically significant (*/#) when the *p*-value was <0.05 (*/#, *p* < 0.05; **/##, *p* < 0.01; ***/###, *p* < 0.001).

## Results

### OCs Increase in the Interface Membrane of Patients With Aseptic Loosening

**FIGURE 1:**
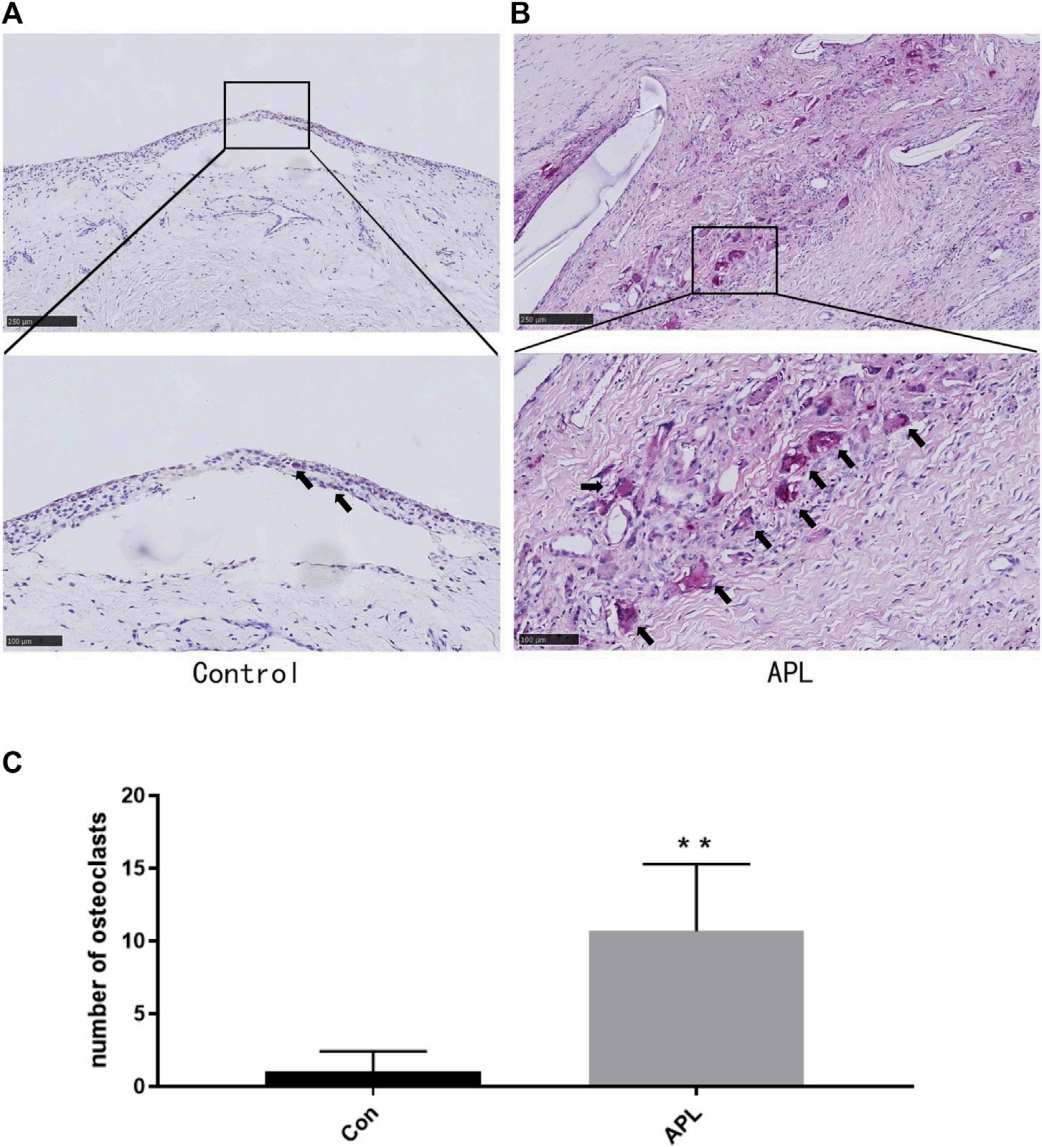
Bone destruction in the interface membrane of patients with aseptic loosening and control group. **(A,B)** Representative TRAP-stained images in the control group **(A)** and aseptic loosening group **(B)**. *p < 0.05 compared with the control group. **(C)** TRAP-positive OCs were quantified, and different data of at least three samples were collected for each group. **p < 0.01 compared with the control group(Con). Image magnification, ×50–200×; scale bar, 100–500 μm.

We collected interface membrane specimens and prepared paraffin sections to investigate bone osteolysis around the prosthesis. OC genesis was detected by TRAP staining. The results revealed that in the synovial specimens (control), a small number of macrophages were observed in the layers of the synovium and around the vessels. However, the number of TRAP-positive cells was low (Figure 1A). Contrastingly, the number of TRAP-positive cells was significantly higher in the interface membrane of the aseptic loosening group compared to the control (*p* < 0.05) (Figures 1B,C).

### SPHK Inhibitors Reduce OC Differentiation and Osteolysis Induced by Wear Particles *In Vivo*

**FIGURE 2:**
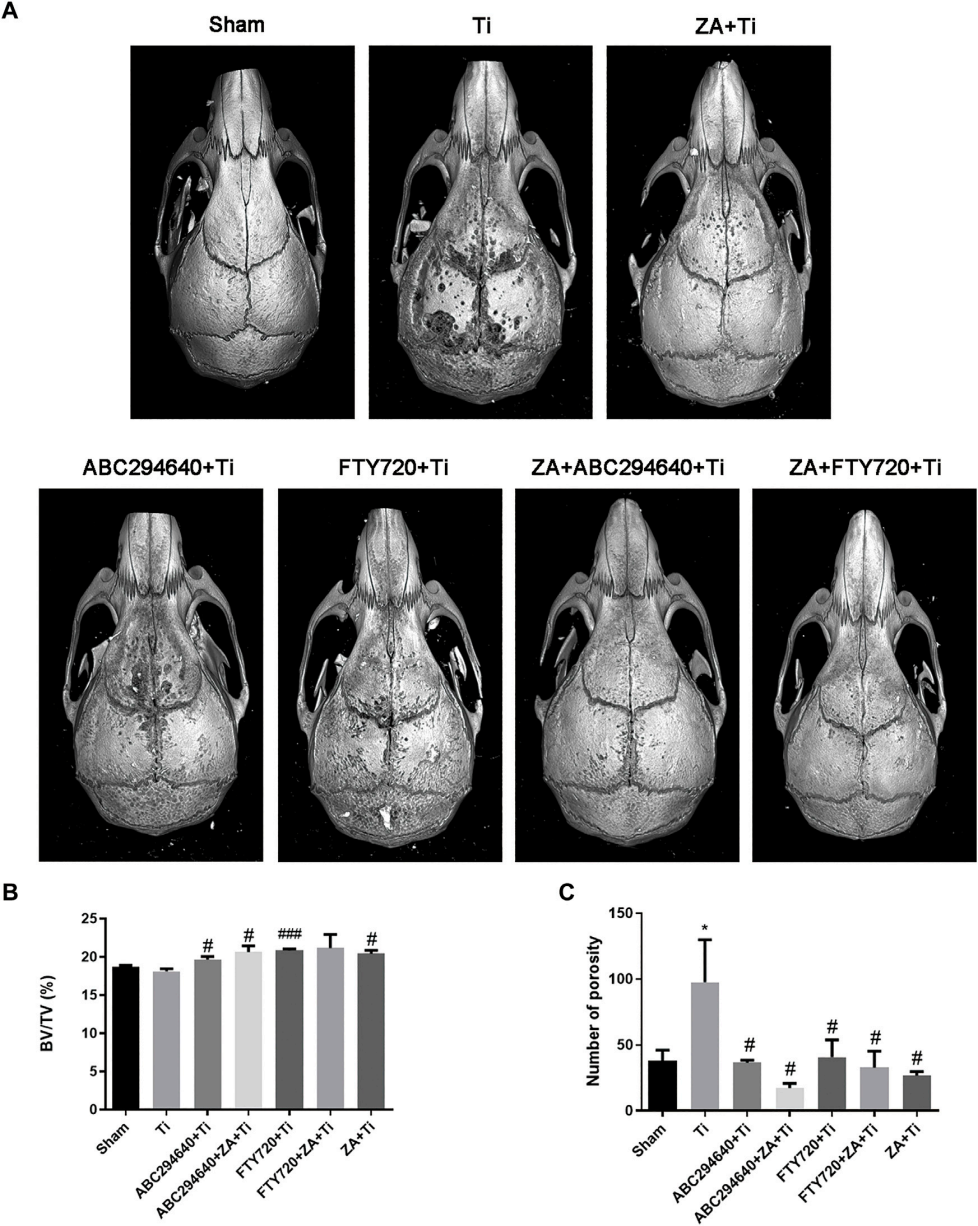
SPHK inhibitors ABC294640 and FTY720 reduce wear particle-induced inflammatory osteolysis in mice. **(A)** Representative image of 3D image reconstruction after micro-CT scan in each group. **(B,C)** Statistical analysis results of the relative bone volume fraction (BV/TV) and the number of pores of the sample ROI. The statistical results were obtained from the collected samples (*n* ≥ 3). *p < 0.05 compared with the Sham group, and #*p* < 0.05 compared with the Ti particle group.

In this study, we verified the functions of SPHK inhibitors in a mouse model of calvarial osteolysis. Micro-CT scanning results showed that bone resorption on the calvarial surface increased (Figure 2A). In addition, the relative bone volume fraction (BV/TV) and the number of pores were markedly higher in the groups treated with SPHK inhibitors (ABC294640 and FTY720) and ZA than in the Ti group (*p* < 0.05) (Figures 2B,C). Moreover, ZA combined with SPHK inhibitors (ABC294640 and FTY720) reduced the degree of wear particle-induced osteolysis and the number of pores in the calvariae when compared with the ZA group (positive control) (*p* < 0.05) (Figure 2).

**FIGURE 3:**
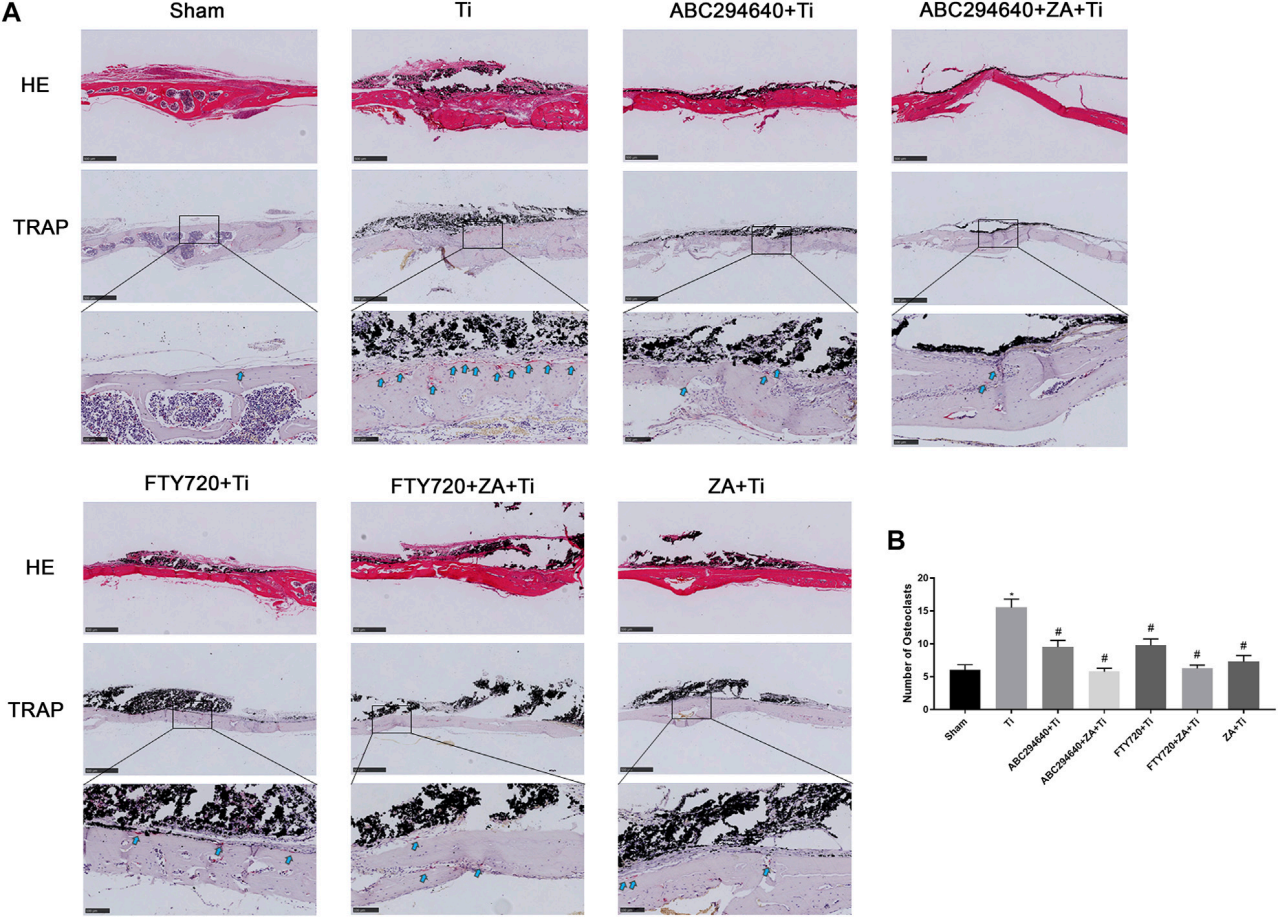
Bone resorption and OC formation were observed in the calvarial tissue specimens of mice stained with TRAP. **(A)** Representative TRAP staining image of each group (×200 magnification); the black arrow shows OCs positive for TRAP staining. **(B)** TRAP-positive OCs were quantified, and different data of at least three samples were collected for each group, which were expressed as the mean ± SEM. *p < 0.05 compared with the Sham group, and #*p* < 0.05 compared with the Ti group. Image magnification, ×50–200×; scale bar, 100–500 μm.

We performed H&E and TRAP staining of the calvarial tissues to assess the morphology and OC formation *in vivo*. Inflammatory infiltrating macrophages and lymphocytes, as well as the proliferation of OCs, were observed in the calvariae of mice stimulated with Ti particles (Figure 3A). TRAP staining showed multinuclear TRAP-positive cells in the eroded cranium lacunae, and the number of OCs was significantly increased in the Ti group. However, the number of TRAP-positive cells was markedly decreased during Ti particle-stimulated osteolysis in mice treated with ABC294640 and FTY720 (Figure 3B). Compared with the single positive control ZA group, ZA combined with SPHK inhibitors ABC294640 and FTY720 reduced the degree of osteolysis and the number of TRAP-positive cells *in vivo* (*p* < 0.05) (Figure 3B). These results show that ABC294640 and FTY720 ZA effectively reduced OC differentiation and the degree of wear particle-induced osteolysis in calvarial bone tissues.

### SPHK Inhibitors and ZA Reduce the Expression of TNF-α and IL-6 *In Vivo*

**FIGURE 4:**
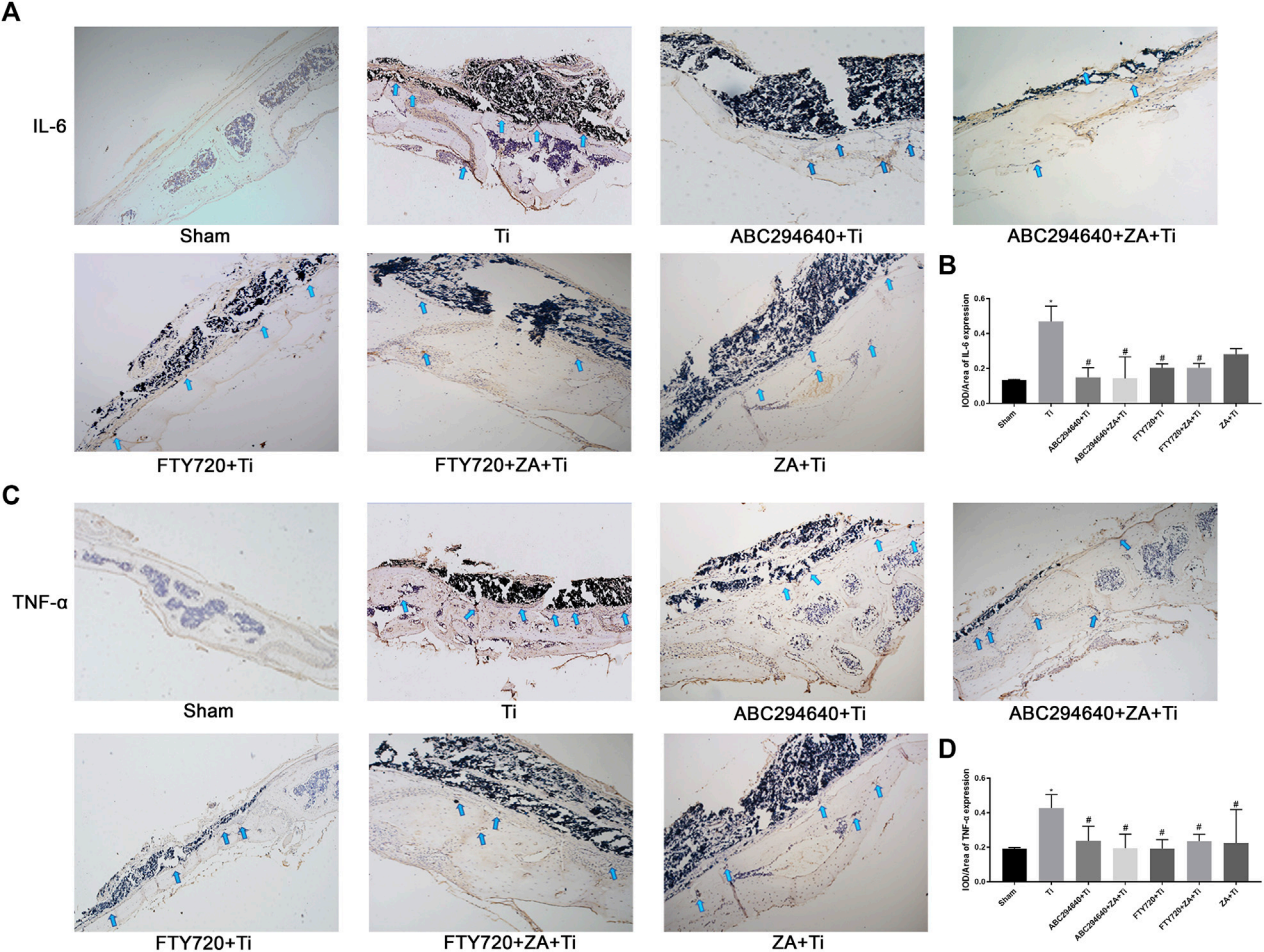
SPHK inhibitors (ABC294640/FTY720) and ZA reduce the expression of pro-inflammatory factors TNF-α and IL-6 in vivo. **(A)** Representative IHC staining images and the for IL-6 in mouse calvariae. **(B)** Quantitative analysis of the expression of pro-inflammatory factor IL-6. **(C)** Representative IHC staining images for TNF-α in mouse calvariae. **(D)** Quantitative analysis of the expression of pro-inflammatory factor TNF-α. Data were collected from samples (*n*≥3) in each group and expressed as the mean ± SEM. *p < 0.05, **p < 0.01 vs. sham group, #p < 0.05, ##p < 0.01 vs. Ti (titanium) group. Image magnification, 100×; scale bar, 200–μm.

We examined the expression of IL-6 (Figure 4A) and TNF-α (Figure 4C) around calvarial tissue stimulated by Ti particles to investigate the effect of SPHK inhibitors (ABC294640/FTY720) and ZA on the expression and secretion of inflammatory cytokines. However, after the intraperitoneal injection of ABC294640, FTY720, and ZA, upregulation of the inflammatory factors stimulated by Ti particles was inhibited (Figures 4B,D). These results showed that SPHK inhibitors (ABC294640/FTY720) and ZA significantly reduced the TNF-α and IL-6 inflammatory cytokines in inflammatory osteolysis induced by wear particles.

### SPHK Inhibitor (ABC294640) and ZA Inhibited the Expression of OC-Related Genes

**FIGURE 5:**
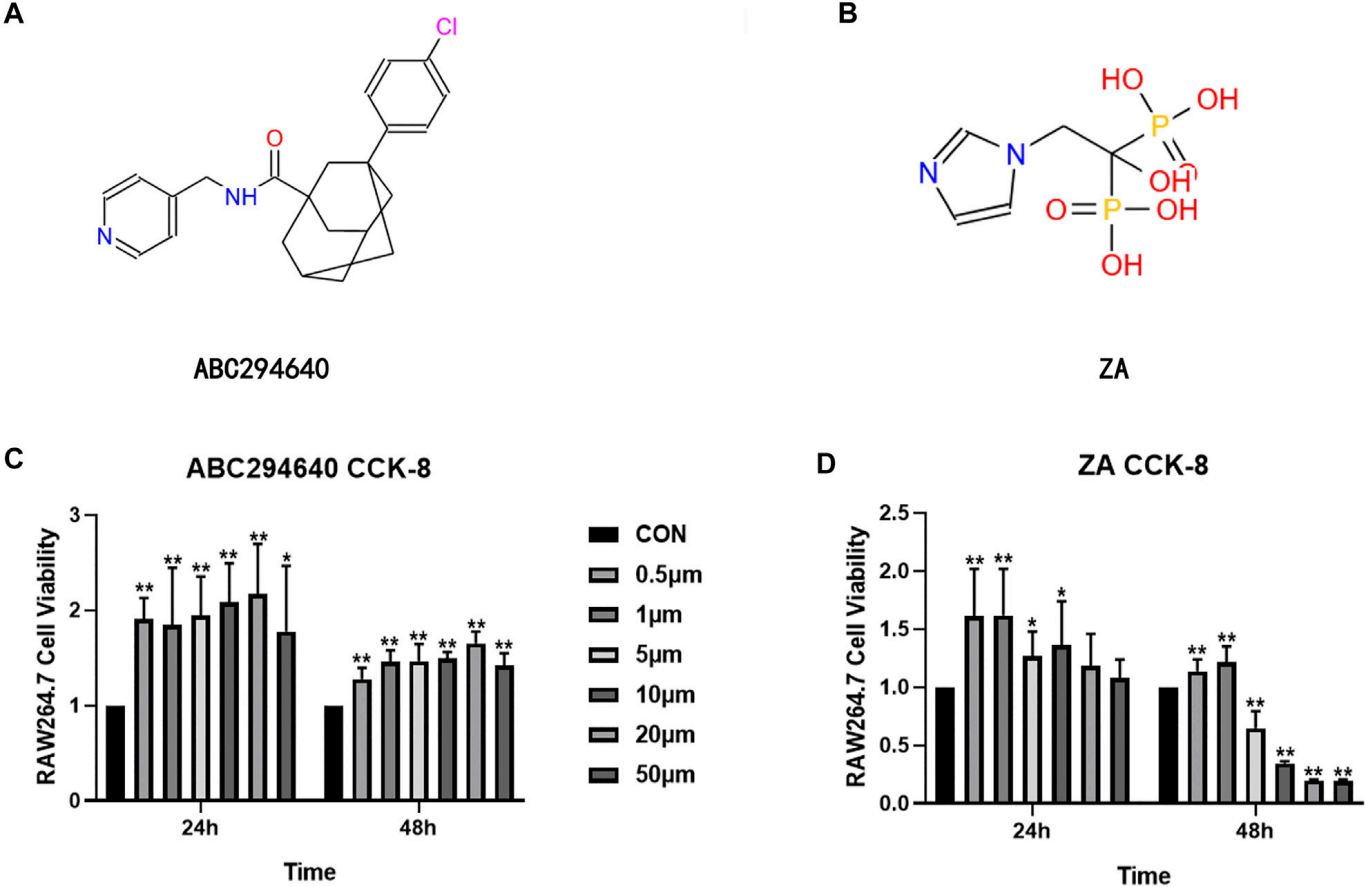
Structural formula of ABC294640 **(A)** and ZA **(B)**. The effects of different concentrations of ABC294640 **(C)** and ZA **(D)** on the viability of RAW264.7 cells were investigated through a CCK-8 experiment. Ratios of OD/OD of the NC group are shown as means ± SEM. *p < 0.05, **p < 0.01 versus the NC group.

**FIGURE 6:**
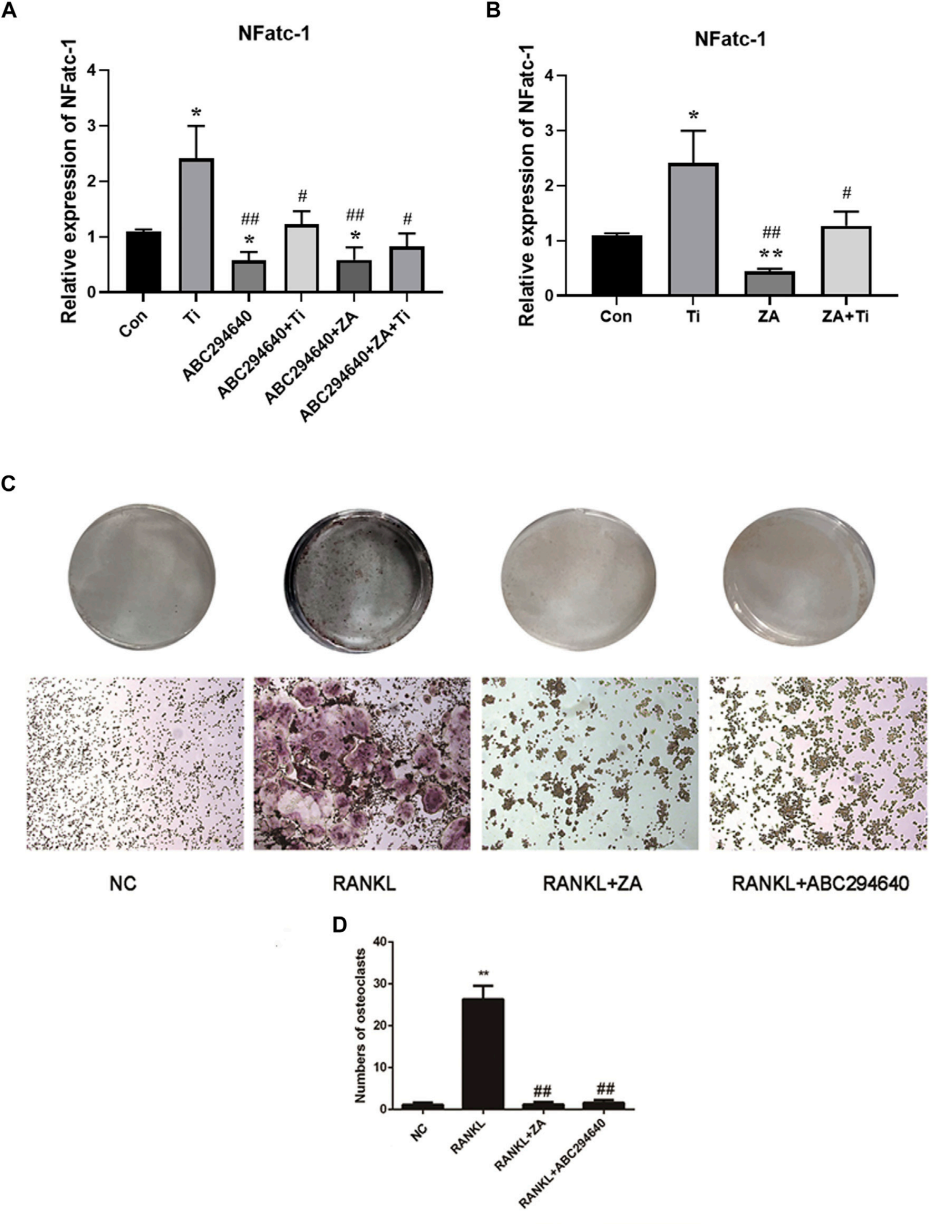
ABC294640 and ZA decrease the mRNA expression of the RANKL-induced genes NFATc-1 and suppress the differentiation and function of OCs. **(A)** RT-qPCR analysis was performed to test the mRNA expression of the OC-related genes NFATc-1 after treating SPHK inhibitors ABC294640 and ZA with Ti particles. **(B)** mRNA expression of the NFATc-1 after the ZA treatment with Ti particle (data from three test experiments are shown as the mean ± SEM; *p < 0.05, **p < 0.01 versus control groups; #*p* < 0.05). **(C**, **D)** After grouping, the SPHK inhibitor ABC294640 and ZA were treated together with macrophages for 24 h; the blank control group (NC) and RANKL group were set. The differentiation of macrophages into OCs induced by RANKL (30 ng/ml). SPHK inhibitor ABC294640 and ZA were added, and OC formation was detected by TRAP staining on the fifth day **(C)**. The number of positive OC cells was counted, and it decreased significantly after treatment with of ABC294640 and ZA (compared with the control + Ti groups; compared with the NC group *p < 0.05; compared with the RANKL group #*p* < 0.05, ##*p* < 0.01). Image magnification, ×100–200×; scale bar, 250–500 μm.

We further evaluated the suppression effect of the drugs on wear particle-induced osteolysis. OC differentiation and activation were associated with RANKL, and RT-qPCR was used to assess whether suppressed RANKL stimulated OC-related gene expression. RAW264.7 cells were treated with an induction medium containing Ti/RANKL and cultured with an SPHK inhibitor (ABC294640) and ZA. The CCK-8 test of ABC294640 and ZA was performed at various times and concentrations, and the results showed that the viability of cells was valid in the range of the concentrations used (20 and 1 μM, respectively) (Figures 5C,D). The RT-qPCR results demonstrated that NFATc-1 expression was markedly downregulated by the treatments (*p* < 0.05) (Figures 6A,B). The SPHK inhibitor ABC294640 (Figure 6A) and ZA (Figure 6B) significantly decreased the mRNA levels of NFATc-1 compared with the control group. Therefore, the results confirmed that the SPHK inhibitor (ABC294640) and ZA could inhibit the expression of OC-specific factors during OC differentiation in the APL microenvironment *in vitro*.

### ABC294640 and ZA Suppress the Differentiation and Function of OCs *In Vitro*

To explore the effect of SPHKs and ZA on the macrophage responses induced by wear particles *in vitro*, inflammatory osteolysis was induced by Ti particles, which was inhibited by SPHK inhibitors (ABC294640/FTY720) and ZA. We stimulated RAW264.7 macrophages with Ti particles, used different SPHK inhibitors in groups, and added ZA to induce OC differentiation using RANKL. The TRAP staining results showed the addition of SPHKS inhibitor (ABC294640) and ZA in the positive control group (Figures 6C,D). However, the ratio of integrated optical density to the area of TRAP staining and the number of OCs decreased significantly after treatment with the SPHK inhibitor and ZA (Figure 6D). These results suggest that ABC294640 and ZA effectively suppressed the differentiation and function of RANKL-induced OCs *in vitro*.

### SPHKs Could Mediate the Increase in the Expression Levels of S1P, TRAF2, and BECN1 Exposed to Ti Particles

**FIGURE 7:**
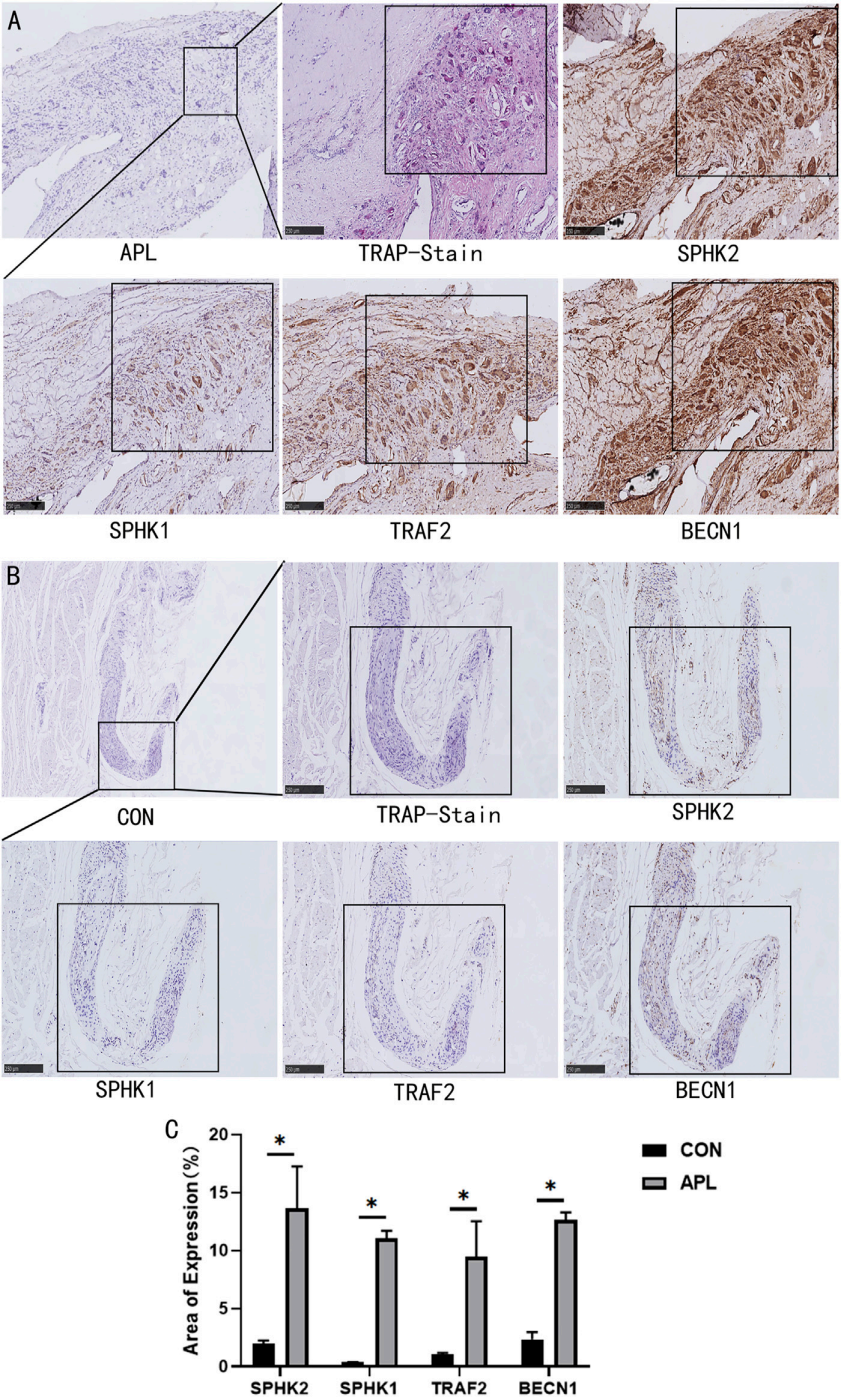
SPHKs mediate osteolysis with TRAF2-BECN1. **(A, B)** Representative positive IHC staining and TRAP staining images of APL and Control specimen groups. **(C)** Quantitative analysis of the expression of SPHK1, SPHK2, TRAF2, BECN1 proteins. Data were collected from samples in each group and expressed as the mean ± SEM. *p < 0.05, **p < 0.01 vs. control group (CON). Image magnification,100×~200×; scale bar, 250~500 μm.

Recent studies have revealed that S1P and BECN1 are key mediators of OC genesis. Furthermore, previous studies have revealed that SPHKs can regulate autophagy through the SPHKs/S1P-TRAF2-BECN1 signaling pathway (Schenten et al., 2011; Ruspi et al., 2014; Liu et al., 2017; Liang et al., 2020). To investigate the osteolysis and expression of SPHKs-TRAF2-BECN1 around the prosthesis, we collected interface membrane tissue specimens and prepared paraffin sections. OC genesis was detected by TRAP staining, and IHC detected the expression of SPHKs-TRAF2-BECN1 proteins (Figure 7A–C). Our results showed that many macrophages scattered in the synovium layers and around vessels in the aseptic loosening specimens. In addition, the number of TRAP-positive cells was high (Figures 7A,B), whereas the SPHKs-TRAF2-BECN1 expression was high in the same location (Figures 7A,B), thus suggesting that under the stimulation of Ti particles, SPHKs can induce macrophage autophagy through the mediated S1P-TRAF2-BECN1 pathway, providing a theoretical basis for studying the role and mechanism of SPHKs in the stimulation of inflammation and osteolysis by wear particles.

**FIGURE 8:**
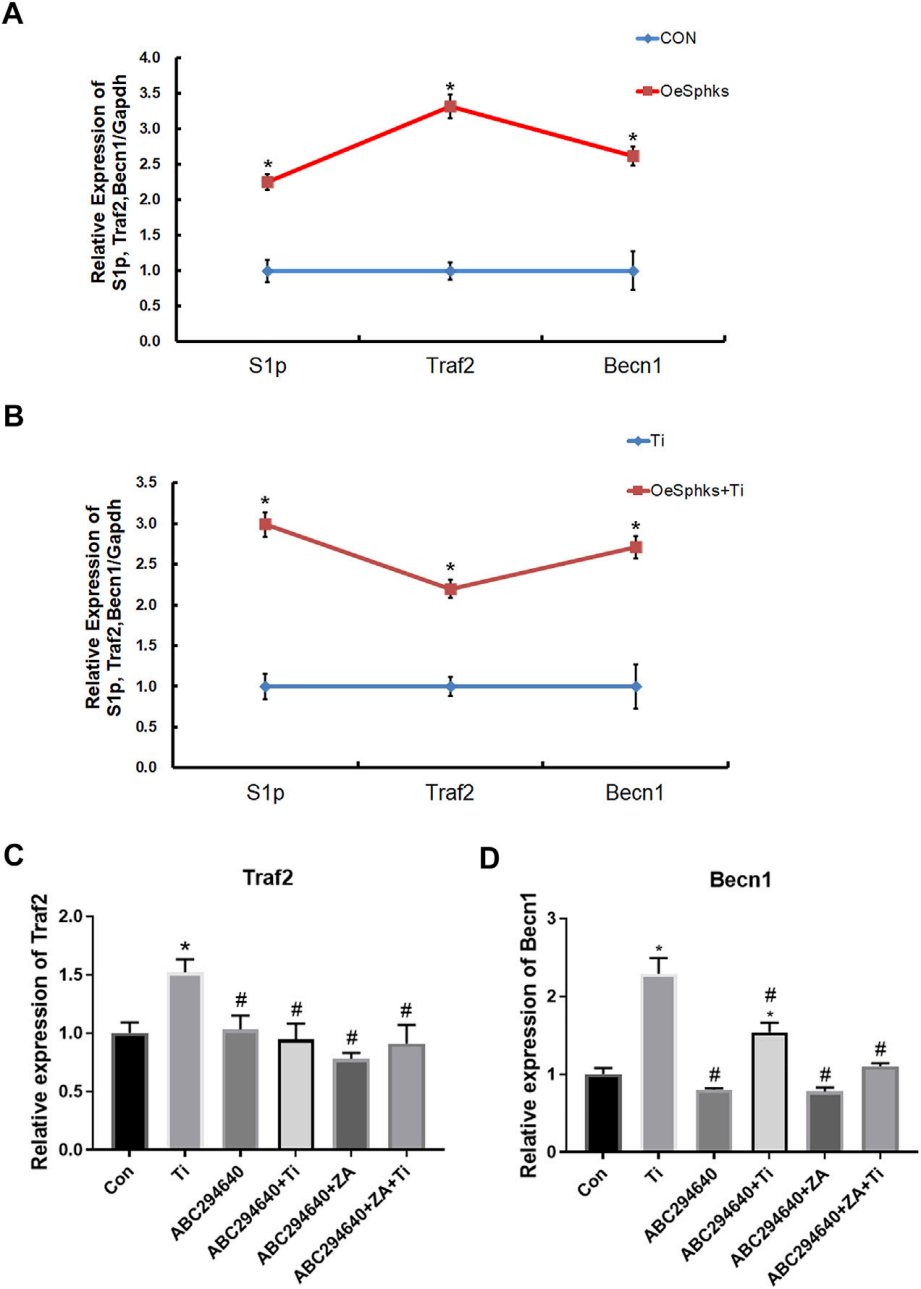
SPHKs mediate Ti-induced osteolysis via S1P-TRAF2-BECN1. **(A**, **B)** Expression of S1P, TRAF2, and BECN1 genes in macrophages overexpressing SPHKs by RT-qPCR 24 h after grouping treatment, treated with Ti. **(C, D)** Ti was used to stimulate the macrophages 24 h in ABC294640/ZA groups, and then the expression of Traf2 and Becn1 genes was evaluated by RT-qPCR. Data from at least three isolated experiments are shown as the mean ± SEM. *p < 0.05, **p < 0.01 versus control groups.

To clarify the role of the S1P-TRAF2-BECN1 pathway mediated by SPHKs in wear particle-induced inflammatory osteolysis, we constructed vector transfection to overexpress SPHKs in RAW264.7 macrophages (Supplementary Materials 1 and 2), treated them with Ti particles in groups, and analyzed the relevant results. The results showed that the expression levels of S1P, TRAF2, and BECN1 genes in macrophages overexpressing SPHKs were increased compared with the control group (Figures 8A,B). Furthermore, the expression levels of TRAF2 and BECN1 were decreased by ABC294640 and ZA in the groups with Ti particles (Figures 8C,D).

## Discussion

Artificial joint replacement has become one of the most effective methods for treating severe joint trauma, inflammation, and other end-stage joint diseases (Learmonth et al., 2007; Gu et al., 2015). The number of patients undergoing artificial hip and knee replacement surgery has exceeded two million per year worldwide (Sarode et al., 2016; Ferguson et al., 2018). Prosthesis loosening is one of the most important complications after artificial joint replacement and causes prosthesis failure. However, there are no specific prevention and treatment drugs, leaving patients to undergo revision surgery or multiple operations. Therefore, it is of clinical significance and educational value to investigate the internal mechanism of the occurrence and development of aseptic loosening of joint prostheses and explore the corresponding molecular targets, new potential drugs, and treatment strategies.

The mechanism of occurrence and development of prosthesis aseptic loosening has not been investigated. Therefore, there are no effective prevention and treatment targets. The mechanisms of action that have been confirmed include interface fretting, prosthesis wear, wear particle stimulation, systemic and local inflammatory responses, immune status changes after implantation, and inflammatory osteolysis induced by wear particles (McEvoy et al., 2002; Goodman and Gallo, 2019). A previous study found that prosthetic wear particles induce inflammatory osteolysis of macrophages, which is the main reason for the aseptic loosening of prostheses (Goodman et al., 2014; Pajarinen et al., 2014). The main components of the joint prosthesis are made of metal materials. Furthermore, the wear particles generated by the friction between the components after joint replacement are released at the bone–implant interface and pass through toll-like receptors (TLRs), activating the migration and polarization of macrophages, continuously releasing pro-inflammatory substances such as TNF-α and IL-6, activating OCs, causing inflammatory osteolysis, and ultimately leading to the occurrence of prosthesis loosening (Mbalaviele et al., 2017; Camuzard et al., 2019). Our previous study confirmed that persistent stimulation with Ti particles leads to a consistent release of TNF-α and IL-6 through SPHK activity, leading to aseptic implant loosening. However, SPHK inhibitors can markedly reduce inflammatory cytokines. However, according to previous studies, S1P and BECN1 are key mediators of OC genesis. Treatment with FTY720 suppressed mature OC formation and attenuated ovariectomy-induced osteoporosis (Ishii et al., 2009). Thus, appropriate regulation of SPHKs may be a new treatment of inflammatory osteolysis and aseptic implant loosening.

Our previous study showed that SPHKs are an important mediator of the regulation of wear particles to induce macrophage inflammation. Ti particles can activate the synthesis and release of TNF-α and IL-6 in macrophages through SPHKs, leading to local long-lasting chronic inflammation and damage to native immune function, ultimately leading to prosthesis loosening (Yang et al., 2018). However, the role of SPHKs in regulating osteolysis and the specific mechanism needs to be investigated.

ABC294640 and FTY720 are classic inhibitors of SPHKs, and ZA is a drug that prevents accelerated periprosthetic bone loss after THA in patients with osteopenia and osteoporosis (Fu et al., 2019). This study found that ZA and SPHK inhibitors reduced inflammation and Ti particle-induced prosthesis loosening in a mouse calvarial osteolysis model. The implant loosening model of the calvarial bone induced by Ti particles is a classical animal model in the study of prosthesis loosening. Our study provides evidence that Ti particles around the prosthesis cause inflammatory cell infiltration and OC proliferation, resulting in increased osteolysis, leading to APL. Our findings correspond with previous studies indicating that inhibiting OC formation and inflammation mitigates wear particle-induced implant loosening (Shin et al., 2020). This result provides a theoretical basis for SPHK inhibitors to become a potential therapeutic drug for inflammatory osteolysis and joint prosthesis loosening. In this study, H&E and TRAP staining and micro-CT were performed to assess the function of SPHK inhibitors and ZA *in vivo*. The results showed that the SPHK inhibitors ABC294640 and FTY720 reduced OC differentiation and osteolysis. Furthermore, ZA combined with SPHK inhibitors ABC294640 and FTY720 effectively reduced the degree of osteolysis and the number of pores in the mouse calvariae stimulated by Ti particles compared with the single positive control ZA group (Figures 2–4). This suggests that ABC294640 and FTY720 might be effective in preventing and treating aseptic loosening of prostheses *in vivo*.

However, recent studies have revealed that S1P and BECN1 are key mediators of OC genesis (Ishii et al., 2009; Chung et al., 2014). Previous studies have revealed that SPHKs/S1P and TRAF2 can mediate the complex of BECN1, thereby regulating the flux and level of autophagy (Schenten et al., 2011; Ruspi et al., 2014; Liu et al., 2017; Liang et al., 2020). Ishii et al. found that S1P is a critical control point in OC genesis that may be a therapeutic target by regulating OC precursors and bone mineral homeostasis. Furthermore, treatment with FTY720 suppressed mature OC formation attached to the bone surface and attenuated ovariectomy-induced osteoporosis (Ishii et al., 2009). In addition, Chung et al. reported that BECN1 mediates RANKL-induced OC genesis by regulating the NFATc-1 expression. Currently, there are no reports on the regulation of inflammatory osteolysis in prosthetic loosening by wear particles through SPHKs and related signaling pathways (Chung et al., 2014).

To explore the effect of inhibiting SPHKs on osteolysis induced by wear particles *in vitro*, we stimulated RAW264.7 macrophages with Ti particles and used different SPHK inhibitors to group macrophages and added ZA to induce OC differentiation. OC differentiation and activation were associated with RANKL. The expression of OC genes (NFATc-1) was significantly downregulated by the treatments (*p* < 0.05) (Figures 6A,B). Furthermore, we found that the addition of ABC294640 and ZA in the positive control group significantly inhibited the differentiation of OCs compared with the control group (Figures 6C,D). Thus, the results confirmed that SPHK inhibitors combined with ZA inhibit the expression of OC-specific factors during OC differentiation in the APL microenvironment *in vitro*.

**FIGURE 9:**
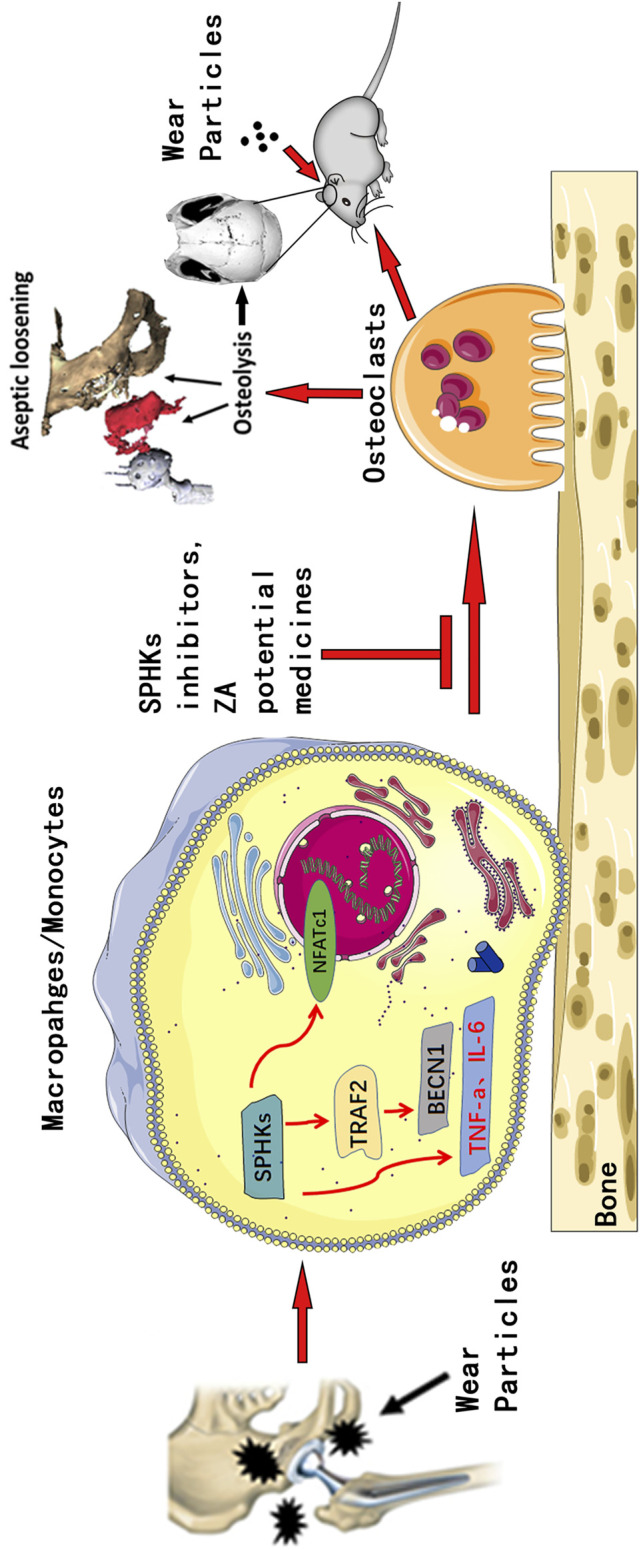
Pattern diagram of SPHKs in modulating OC genesis. SPHKs could induce osteolysis induced by wear particles. SPHK inhibitors and ZA repressed the expression of TRAF2, BECN1, and OC-specific genes to inhibit OC formation and functions.

To further clarify the role of the SPHKs/S1P-TRAF2-BECN1 signaling pathway mediated by SPHKs in wear particle-induced inflammatory osteolysis, we observed a high number of macrophages in the layers of the synovium and around the vessels, and the number of TRAP-positive cells was higher than that in the aseptic loosening specimens (Figure 1, Figures 7A,B). In contrast, the SPHKs-TRAF2-BECN1 expression was higher (*p* < 0.05) (Figures 7C). Furthermore, we selected SPHK-overexpressed RAW264.7 macrophages to be treated with Ti particles in groups. We found increased expression levels of S1P, TRAF2, and BECN1 in the SPHK-overexpressed groups or the groups treated with Ti. However, the expression levels of TRAF2 and BECN1 were decreased by ABC294640 and ZA in groups with Ti particles (Figure 8). The results suggest that under the stimulation of Ti particles, SPHKs can induce macrophage osteoclastogenesis through the SPHKs/S1P-TRAF2-BECN1 pathway, providing a theoretical basis for the study of the role and mechanism of SPHKs in the stimulation of inflammation and osteolysis by wear particles (Figure 9).

However, there are some limitations to our study. First, we found that SPHK inhibitors suppressed OC formation to reduce osteolysis in inflammatory osteolysis and APL induced by wear particles. SPHKs are considered regulators of autophagy. Recently, macrophage autophagy or apoptosis has been related to osteolysis and APL processes. However, whether SPHKs can improve cell autophagy and apoptosis caused by wear particles in inflammatory osteolysis needs to be clarified. In addition, the relation and mechanisms are not known, hence the continued research around this subject. Second, although the mouse calvarial osteolysis model has been broadly used to study the mechanism of aseptic loosening, the bone affected by the Ti particles is a flat bone rather than a long bone, and the period of Ti particle-induced inflammatory osteolysis we studied was not continuous and lasted for only 14 days. Thus, it is necessary to establish a larger animal model of APL, such as primates, which are more closely related to humans. In addition, it is necessary to assess the effectiveness and safety of drug interventions in the long term.

## Conclusion

In conclusion, we demonstrated that wear particles could induce inflammatory osteolysis and prosthetic loosening via upregulating SPHKs expressions and activating SPHKs/S1P-TRAF2-BECN1; SPHK inhibitors combined with ZA suppressed osteoclastogenesis and inflammatory osteolysis, providing a fundamental theoretical basis for the potential treatment of inflammatory osteolysis and prosthesis loosening.

## Data Availability

The original contributions presented in the study are included in the article/Supplementary Material, further inquiries can be directed to the corresponding authors.

## Abbreviations

APL, Aseptic prosthesis loosening; TNF-α, Tumor necrosis factor alpha; IL-6, Interleukin-6; SPHKs, Sphingosine kinases; S1P, Sphingosine-1-phosphate; TRAF2, TNF receptor-associated factor 2; BECN1, Beclin-1; MAPKs, Mitogen-activated protein kinases; OBs, Osteoblasts; OCs, Osteoclasts; RANKL, Receptor activator of nuclear factor-kappa B ligand; mROS, Mitochondrial reactive oxygen species; NFATc-1, Nuclear factor of activated T-cell cytoplasmic 1; ZA, Zoledronic acid; THA, Total hip arthroplasty; TRAP, Tartrate-resistant acid phosphatase; TLRs, Toll-like receptors.
