# Supplementary material for: SPHK Inhibitors and Zoledronic Acid Suppress Osteoclastogenesis and Wear Particle-Induced Osteolysis
Source: Front Pharmacol. 2022 Feb 14;12:794429. doi: 10.3389/fphar.2021.794429 (PMC8883393; doi:10.3389/fphar.2021.794429)
Supplement: Supplementary file 1 [file DataSheet1.docx]

**Supplemental Materials**

Supplemental material 1


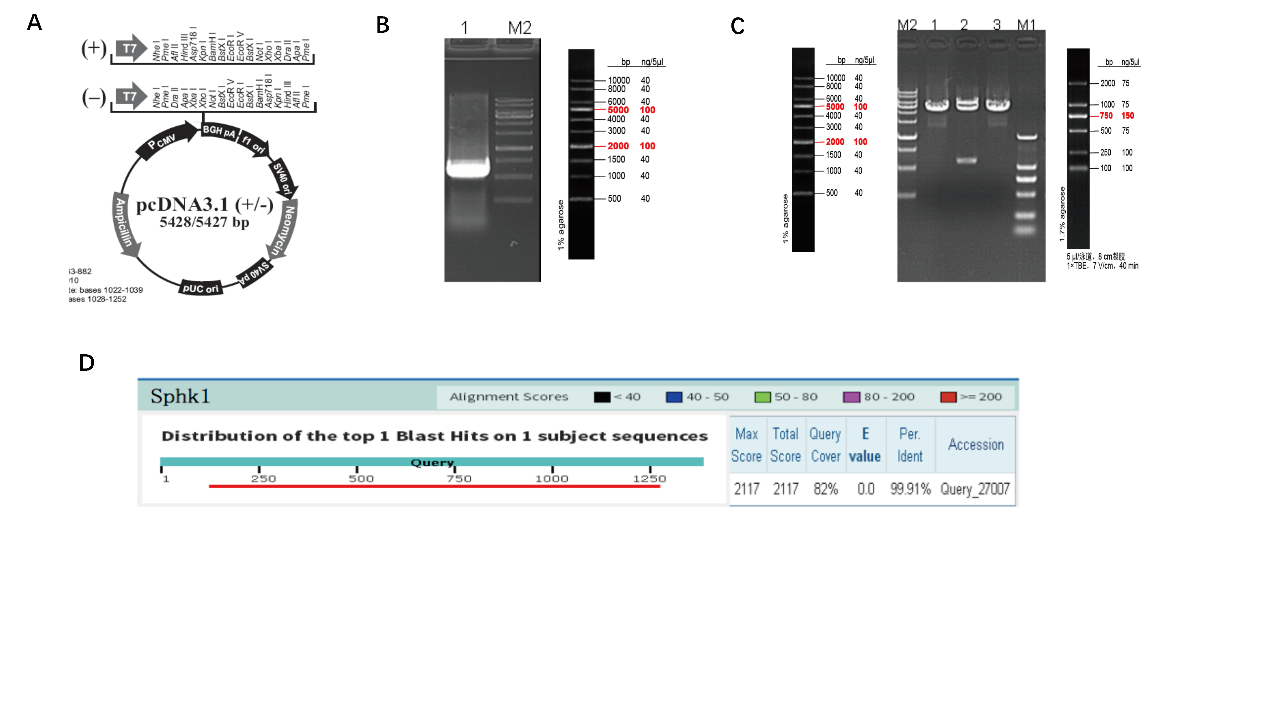


Supplemental material 1

**A** The information of the vector we used. **B** Lane M2: 1kb DNA Marke; Lane 1: SPHK1 PCR amplification product (1149bp); 5μl/lane, 8cm gel, 1xTAE, 7V/cm, 45min, SPHK1 has been amplified with the template, and the size is consistent with the prediction (The amplified band is approximately one point on the Marker 1000bp); SPHK1 target gene has been amplified. **C** Lane M2: 1 kb DNA Marker; Lane 1: Respectively the products after the plasmid digestion of SPHK1-1; Lane 2: Respectively the products after the plasmid digestion of SPHK1-2; Lane 3: Respectively the products of SPHK1-3 after plasmid digestion; Lane M1: DL2000 DNA Marker; Enzyme digestion results showed that SPHK1 (1149bp) cuts out a band of interest (pointed by the red arrow) at the corresponding position. **D** Sequencing results BLAST analysis: SPHK1 has been successfully cloned into the pcDNA3.1+ vector, which is 99% consistent with the known sequence on NCBI by BLAST.

Supplemental material 2


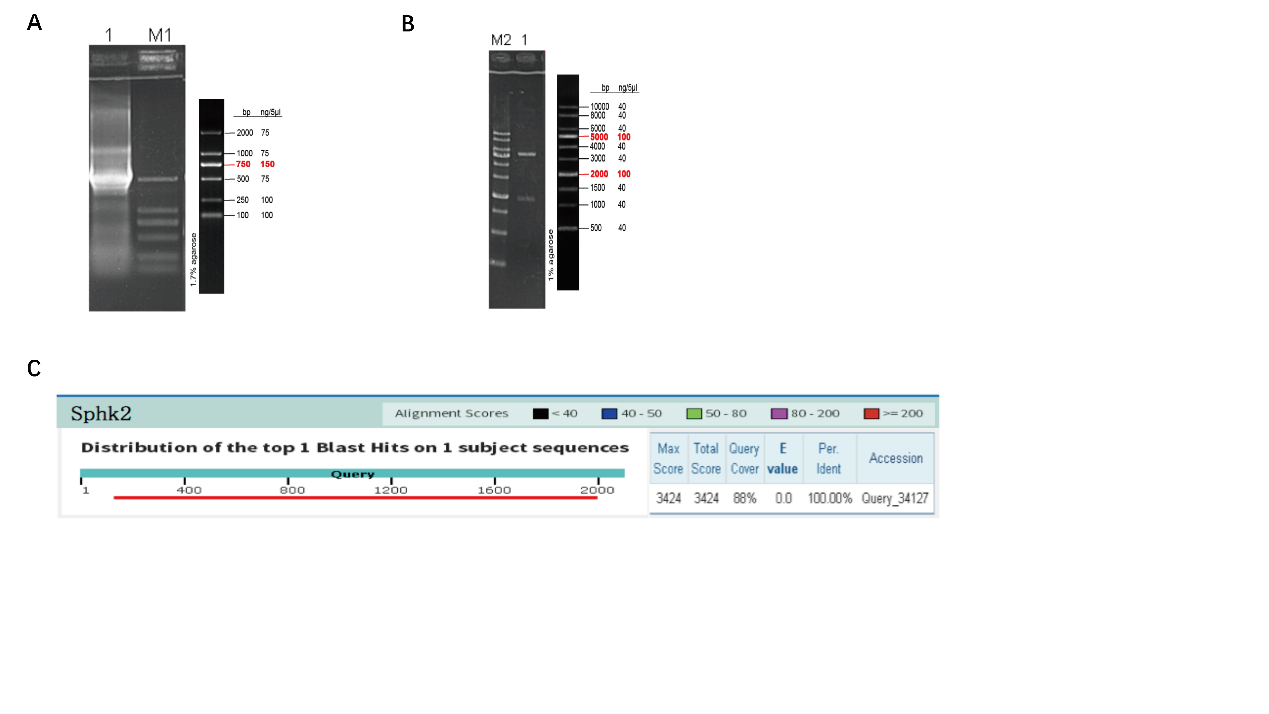


Supplemental material 2

**A** Lane M1: DL2000 DNA Marker; Lane 1: SPHK2 PCR amplification product (1854bp); Result analysis: SPHK2 has been amplified using the template, and the size is consistent with the prediction (the amplified band is about a point below the Marker2000bp); it proves that the SPHK2 target gene has been amplified. **B** Lane M2: 1 kb DNA Marker; Lane 1: Respectively the products after plasmid digestion of SPHK2-1; Analysis of enzyme digestion results: SPHK2 (1854bp) cuts out a band of interest (pointed by the red arrow) at the corresponding position, indicating that a positive clone has been screened, and the SPHK2 positive plasmid is sent for sequencing. **C** BLAST analysis of sequencing results: SPHK2 has been successfully cloned into pcDNA3.1+ vector, which is 100% consistent with the known sequence on NCBI by BLAST, so it can be used in subsequent experiments.
